# Prognostic analysis of different postoperative adjuvant therapies for patients with hepatocellular carcinoma after radical resection with high-risk recurrence factors: a multicenter real-world retrospective study

**DOI:** 10.3389/fimmu.2025.1661923

**Published:** 2025-10-22

**Authors:** Zejin Zhao, Yue Xiao, Chen-guang Su, Hui Zhao, Jian Li, Jinlong Liu

**Affiliations:** ^1^ Department of Hepatobiliary Surgery, The Affiliated Hospital of Chengde Medical University, Chengde, Hebei, China; ^2^ Department of Thoracic Surgery, The Second Hospital of Hebei Medical University, Shijiazhuang, Hebei, China; ^3^ Department of Gastrointestinal Surgery II, Tangshan People’s Hospital, Tangshan, Hebei, China; ^4^ Hebei Key Laboratory of Panvascular Diseases, Chengde, Hebei, China

**Keywords:** hepatocellular carcinoma, high-risk recurrence, postoperative adjuvant therapy, propensity matching analysis, real-world study

## Abstract

**Background:**

Hepatocellular carcinoma (HCC) is one of the most common malignant tumors worldwide, with high postoperative recurrence rates significantly limiting long-term survival, particularly in patients with high-risk features such as large tumor diameter (≥5 cm), multiple tumors (≥3 nodules), microvascular invasion (MVI), or portal vein tumor thrombus (PVTT). There is still considerable controversy about the efficacy of adjuvant therapy after liver resection (LR) in improving the prognosis of HCC patients with high risk of recurrence and its therapeutic efficacy in different high-risk subgroups.

**Materials and methods:**

This multicenter retrospective study included 300 patients with high-risk HCC who underwent liver resection in four medical institutions in China from January 2015 to April 2024, including 101 patients in the LR group and 199 patients in the LR+ postoperative adjuvant therapy group.

**Results:**

During follow-up, 178 patients (59.3%) died. OS was significantly better in the LR plus adjuvant therapy group than in the LR alone group (entire cohort: HR = 0.55, 95% CI: 0.39–0.76, P<0.001; matched cohort: HR = 0.47, 95% CI: 0.32–0.71, P<0.001). The median OS in the matched cohort was 32.1 months (95% CI: 25.4-38.8) for the adjuvant group versus 18.5 months (95% CI: 14.2-22.8) for the LR group. In the matched cohort, 1-, 3-, and 5-year OS rates were 82.2%, 46.0%, and 20.2% for the adjuvant group versus 63.4%, 33.1%, and 17.2% for the LR group. DFS was also significantly prolonged in the adjuvant group (HR = 0.43, 95% CI: 0.29–0.65, P< 0.001), with a median DFS of 15.3 months (95% CI: 11.9-18.7) compared to 8.1 months (95% CI: 6.5-9.7) in the LR group, and 1-year DFS of 53.4% vs 30.9%. Multivariate analyses identified AFP, ALB, tumor diameter, PVTT, TACE, and adjuvant therapy as independent predictors of OS, while AFP, multiple tumors, MVI, PVTT, TACE, and adjuvant therapy were associated with DFS. Subgroup analysis showed that TACE offered significant benefit (OS: HR = 0.54; DFS: HR = 0.55), and TKI therapy also demonstrated improved outcomes (OS: HR = 0.58; DFS: HR = 0.58).

**Conclusion:**

Postoperative adjuvant therapy provides significant survival benefits for HCC patients with high risk of recurrence, with TACE therapy demonstrating the greatest efficacy. These findings underscore the importance of individualized postoperative management in this population.

## 1 Introduction

Hepatocellular carcinoma (HCC) is one of the most common malignant tumors in the world and the leading cause of cancer-related death worldwide (1). Radical surgical resection is the basis for the treatment of HCC patients (2). Although modern surgical techniques have made great progress, the long-term survival rate of patients is still significantly restricted by the high recurrence rate after surgery, which is particularly significant in patients with high-risk factors such as huge tumor volume (≥ 5 cm), multiple tumors (number ≥ 3), microvascular invasion (MVI) and portal vein tumor thrombus (PVTT) (3, 4). These high-risk recurrence factors together reflect the biological characteristics of invasive tumors, and are closely related to early recurrence and poor prognosis of patients. There is an urgent need for effective postoperative adjuvant therapy to improve the prognosis of patients with high-risk recurrence.

In current clinical practice, adjuvant therapy for HCC mainly includes transcatheter arterial chemoembolization (TACE), hepatic arterial infusion chemotherapy (HAIC) and tyrosine kinase inhibitors (TKIs) (5). Although existing studies have shown that systemic adjuvant therapy can significantly improve the prognosis of patients, there is still a lack of consensus on standardized adjuvant therapy after radical resection of liver cancer. For example, although there is evidence that adjuvant TACE can reduce the risk of recurrence by removing residual micrometastases after surgery (6–9), studies have shown that its survival benefits are limited, especially in high-risk subgroups with MVI or PVTT (10). Similarly, although TKIs such as sorafenib and lenvatinib have been proved to be effective in advanced HCC (11–13), the results of different studies on the same postoperative adjuvant therapy are inconsistent, which may be related to the differences in patient screening criteria and treatment tolerance (14, 15). These controversies highlight the importance of accurately identifying patients with high risk of recurrence and formulating individualized adjuvant treatment strategies, and achieving real survival benefits.

This study retrospectively analyzed the clinical data of multiple centers to evaluate the effect of postoperative adjuvant therapy on the overall survival (OS) and disease-free survival (DFS) of HCC patients with high-risk recurrence factors after hepatectomy. By using propensity score matching (PSM) to reduce selection bias and conducting stratified analysis on key prognostic factors including AFP level, tumor diameter, tumor number, MVI and PVTT, we aim to clarify the efficacy of adjuvant therapy in different high-risk recurrence risk subgroups. Our results provide evidence for optimizing individualized treatment and postoperative adjuvant therapy for HCC patients with high risk of recurrence.

## 2 Methods

### 2.1 Study population

HCC patients who underwent LR radical resection in four medical centers in China (Affiliated Hospital of Chengde Medical College, Tangshan Cancer Hospital, Tangshan Workers’ Hospital and Tangshan Kailuan General Hospital) from January 2015 to April 2024 were retrospectively studied. Inclusion criteria (1): pathological diagnosis of HCC and meet at least one of the following high-risk recurrence factors: a) MVI or PVTT; b) The maximum tumor diameter≥ 5cm; c) The number of tumors≥ 3 (2); Radical hepatectomy. Exclusion criteria (1): age < 18 years (2); Recurrent HCC or synchronous extrahepatic metastasis (3); receiving preoperative anti-cancer treatment (4); have a history of other malignant tumors (5); Postoperative immunotherapy or combination therapy (6); Missing key variables in clinical data (7); Perioperative death (within 30 days after surgery).

### 2.2 Data collection and variables

Demographics, clinical features, laboratory findings, imaging and pathological features, surgical data, adjuvant therapy and follow-up data were retrospectively collected from the medical record system of each medical center. The following variables were analyzed: age, gender, hepatitis virus infection status, maximum tumor diameter, number of tumors, presence of microvascular invasion, presence of portal vein tumor thrombus, blood loss, blood transfusion, preoperative AFP, ALT, AST, ALB, TBIL and postoperative adjuvant therapy. Given the small sample size of the HAIC subgroup (n=9), the primary analysis focused on comparing the LR group, the TACE group (n=141), and the TKI group (n=49). Propensity score matching (PSM) was used to balance baseline characteristics. Kaplan-Meier method and Cox regression model were used to evaluate overall survival (OS) and disease-free survival (DFS). The proportional hazards assumption for Cox models was verified using Schoenfeld residuals.

### 2.3 High-risk recurrence factors

The clinical definition of high-risk recurrence factors: 1. Multiple tumors: There are≥ 3 independent primary lesions in the liver at the same time, and there is no direct anatomical correlation between the lesions, which are confirmed as primary hepatocellular carcinoma by pathology; 2. Tumor diameter≥ 5cm: The maximum diameter of a single tumor≥ 5cm was measured by preoperative imaging or postoperative pathology; 3. MVI: Postoperative pathology showed that the tumor cells invaded the portal vein, hepatic vein or capillary branches under the microscope, and the invasion range was limited to the vascular endothelial layer. 4. PVTT: Preoperative imaging or postoperative pathology found that the tumor invaded the portal vein system and formed a visible cancerous thrombus in the lumen.

### 2.4 LR and postoperative adjuvant therapy

The liver resection is performed by experienced surgeons. The surgical plan is based on the number, size, location of the tumors, and the patient’s liver function. All liver resections are radical, meaning they involve the complete removal of all detected tumors, with histopathological confirmation of negative margins. The distance from the nearest tumor edge to the resection margin was ≥1 cm.

TACE: 4–6 weeks after the liver resection, once the patient’s liver function has recovered, TACE treatment is administered. The procedure involves using Seldinger technique to puncture the femoral artery and perform hepatic arteriography through a 5 Fr catheter (RH/Yashiro type) to locate the active tumor lesion. Embolic emulsion is slowly injected into the hepatic artery, containing 30- 60mg of anthracyclines (epirubicin, doxorubicin, or pirarubicin), 30- 50mg of platinum-based drugs (epirubicin, cisplatin, or carboplatin), and 5–10 ml of iodized oil. The amount of iodized oil and chemotherapy drugs is determined based on the patient’s liver function and body surface area.

HAIC: For patients with high risk of recurrence, HAIC adjuvant therapy was initiated at 3–4 weeks after surgery. FOLFOX regimen (oxaliplatin 85 mg/m^2^, calcium folinate 400 mg/m^2^ combined with 5-fluorouracil 400 mg/m^2^ injection+ 2400 mg/m^2^ continuous infusion) was continuously infused through interventional catheter in the proper hepatic artery indwelling catheter for chemotherapy. The treatment cycle was once every 3 weeks.

TKI: Oral targeted drug maintenance therapy was started 4 weeks after operation. Conventional regimens include sorafenib (400 mg bid) or lenvatinib (8–12 mg qd). During the treatment, hand- foot skin reaction, proteinuria and thyroid function indexes were monitored every 4 weeks. When ≥ grade 2 adverse reactions occurred, the dose was reduced by 20%- 50% gradient. At the same time, antihypertensive drugs and local skin care were combined for symptom management.

The allocation of patients receiving postoperative adjuvant therapy (TACE, HAIC or TKI) was based on the comprehensive judgment of clinicians’ experience and the preference of patients.

### 2.5 Outcome and definition

The primary endpoint of the study was OS, defined as the time from the date of radical resection to the date of death or last follow-up. The secondary endpoint was DFS, defined as the time from surgical resection to the first radiological evidence of recurrence (newly detected lesions on contrast-enhanced CT or MRI) or death from any cause, whichever occurred first. After LR, HCC was diagnosed according to preoperative imaging and postoperative histopathological evidence. Cirrhosis is histopathologically defined based on the discovery of resected liver specimens.

### 2.6 Statistical analysis

For the main study group, propensity score matching was used to minimize bias between the two groups. The propensity score was calculated using the following 15 variables: gender, age, hepatitis, cirrhosis, Child-Pugh classification, number of tumors, maximum tumor diameter, blood loss, blood transfusion, preoperative AST, preoperative ALT, preoperative ALB, preoperative TBIL, preoperative AFP, MVI, PVTT. For PSM, the nearest neighbor 1: 1 matching scheme with a caliper value of 0.2 (a commonly recommended value to balance matching quality and sample size retention) is used. The proportional hazards (PH) assumption for the Cox regression models was assessed using Schoenfeld residuals; no significant violations were found (global p-value > 0.05). In the propensity score matching cohort, the OS and DFS of the LR group and the LR+ adjuvant therapy group were compared by log-rank test. Median survival times with 95% confidence intervals are reported. T test and Wilcoxon rank test were used to analyze the comparison of continuous variables with or without normal distribution. Categorical variables were analyzed by χ^2^ and Fisher test. The OS and DFS rates were calculated using the Kaplan-Meier method and compared using the log-rank test. Multivariate analysis was performed using the Cox proportional hazard regression model. A *post-hoc* sample size calculation was performed. Assuming a median OS of 12 months in the control group, an improvement to 18 months (HR = 0.67) was considered clinically meaningful. With α=0.05 and power=80%, the required sample size per group was approximately 90. Our final matched sample size (n=100 per group) meets this requirement. Statistical analysis was performed using SPSS software (version 27.0) and R software (version 4.4.2) for Windows. P< 0.05, significant.

## 3 Results

### 3.1 Baseline characteristics

**Figure 1:**
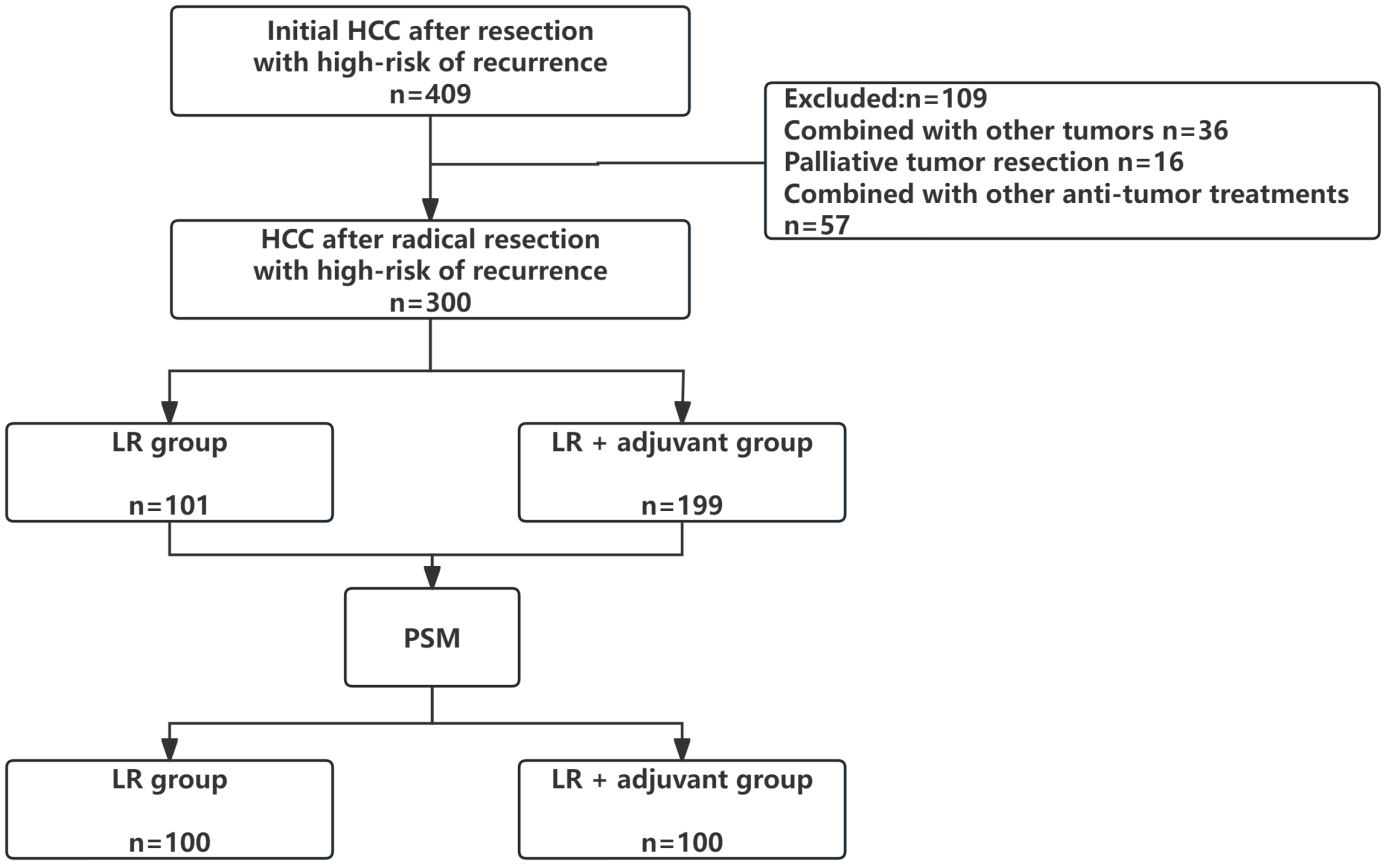
Flow diagram of patient enrollment, exclusion, and grouping.

We reviewed cases from four medical centers between 2015 and 2024, excluding 36 patients with other tumors, 16 patients with palliative tumor resection, and 57 patients with other anti-tumor treatments. The patient selection and grouping process are illustrated in a flow chart (**Figure 1**). Finally, 300 patients were included in this study, and 108 (36.0%) patients were≥ 65 years old. Among them, 234 (78.0%) patients were male. 218 patients (72.6%) had HBV/HCV-related hepatitis. 62 (20.7%) patients had MVI, 53 (17.7%) patients had PVTT, 66 (22%) patients had multiple intrahepatic tumors, 164 (54.7%) patients had huge tumors, 80 (26.7%) patients had high preoperative serum AFP level (defined as serum AFP level≥ 400 ng/ml), 64 (21.3%) patients experienced intraoperative blood transfusion, and 166 (55.3%) patients had cirrhosis. 101 patients (33.7%) only received LR. A total of 199 patients (66.3%) received radical LR and postoperative adjuvant therapy, including 150 patients in the TACE group (n= 141) and the TKI drug treatment group (n= 49). Due to the limited number of patients receiving HAIC (n=9), which precluded meaningful separate statistical analysis, the primary analysis compared three groups: the LR group, the TACE group, and the TKI group. Data for the HAIC subgroup are presented descriptively in the baseline characteristics table (**Table 1**). Patients with TACE regimen received a single TACE treatment, 5–10 ml lipiodol was slowly injected from the hepatic artery, while patients with HAIC regimen received 2–6 times of HAIC treatment, and the HAIC regimen was treated with FOLFOX regimen. The TKI group was treated every 28 days until the recurrence of liver cancer or spontaneous withdrawal. The longest duration of treatment was 2 years. PSM (1: 1 matching) analysis generated two new cohorts of 100 patients in the LR group and the LR+ adjuvant therapy group. The characteristics of the two groups were balanced, and the standardized average difference of all baseline variables was less than 10%. The baseline characteristics of patients in the entire cohort and the matching cohort are summarized in **Table 1**. During the follow-up period, 178 patients died and 168 patients relapsed.

**Table 1:** Baseline characteristics of hepatocellular carcinoma patients with high-risk in different treatment groups.

Characteristics	Entire cohort				Propensity score-matched cohort (1:1 ratio)			
	ALL (n=300)	LR group (n=101)	LR + adjuvant group (n=199)	*P*	ALL (n=200)	LR group (n=100)	LR + adjuvant group (n=100)	*P*
Age				*0.118*				*0.563*
<65	192 (64.0%)	58 (57.4%)	134 (67.3%)		121 (60.5%)	58 (58.0%)	63 (63.0%)	
≥65	108 (36.0%)	43 (42.6%)	65 (32.7%)		79 (39.5%)	42 (42.0%)	37 (37.0%)	
Sex				*0.333*				*0.749*
Male	234 (78.0%)	75 (74.3%)	159 (79.9%)		147 (73.5%)	75 (75.0%)	72 (72.0%)	
Female	66 (22.0%)	26 (25.7%)	40 (20.1%)		53 (26.5%)	25 (25.0%)	28 (28.0%)	
Hepatitis				*0.604*				*1.000*
-	82 (27.3%)	30 (29.7%)	52 (26.1%)		58 (29.0%)	29 (29.0%)	29 (29.0%)	
+	218 (72.7%)	71 (70.3%)	147 (73.9%)		142 (71.0%)	71 (71.0%)	71 (71.0%)	
Cirrhosis				*0.281*				*0.395*
-	134 (44.7%)	50 (49.5%)	84 (42.2%)		93 (46.5%)	50 (50.0%)	43 (43.0%)	
+	166 (55.3%)	51 (50.5%)	115 (57.8%)		107 (53.5%)	50 (50.0%)	57 (57.0%)	
Blood transfusion				*0.755*				*0.188*
No	236 (78.7%)	81 (80.2%)	155 (77.9%)		151 (75.5%)	80 (80.0%)	71 (71.0%)	
Yes	64 (21.3%)	20 (19.8%)	44 (22.1%)		49 (24.5%)	20 (20.0%)	29 (29.0%)	
Bleeding	351 (433)	385 (544)	333 (364)	*0.385*	386 (487)	389 (546)	382 (424)	*0.930*
AFP				*0.133*				*0.408*
<400	220 (73.3%)	80 (79.2%)	140 (70.4%)		152 (76.0%)	79 (79.0%)	73 (73.0%)	
≥400	80 (26.7%)	21 (20.8%)	59 (29.6%)		48 (24.0%)	21 (21.0%)	27 (27.0%)	
ALT	44.0 (52.7)	50.0 (59.9)	40.9 (48.6)	*0.185*	45.6 (54.0)	50.3 (60.2)	41.0 (46.9)	*0.224*
AST	46.7 (55.7)	55.1 (72.3)	42.5 (44.5)	*0.110*	48.2 (59.8)	55.0 (72.7)	41.3 (42.6)	*0.105*
ALB	41.8 (5.91)	41.4 (5.04)	42.1 (6.30)	*0.288*	41.9 (5.77)	41.5 (4.95)	42.4 (6.47)	*0.265*
TBIL	18.2 (18.5)	18.7 (15.7)	17.9 (19.9)	*0.714*	17.6 (12.5)	18.7 (15.8)	16.5 (8.00)	*0.203*
Child-Pugh				*1.000*				*1.000*
A	300 (100%)	101(100%)	199(100%)		200(100%)	100(100%)	100(100%)	
B	0	0	0		0	0	0	
C	0	0	0		0	0	0	
Tumor number				*0.273*				*0.596*
<3	234 (78.0%)	83 (82.2%)	151 (75.9%)		160 (80.0%)	82 (82.0%)	78 (78.0%)	
≥3	66 (22.0%)	18 (17.8%)	48 (24.1%)		40 (20.0%)	18 (18.0%)	22 (22.0%)	
Tumor diameter				*0.575*				*1.000*
<5cm	136 (45.3%)	43 (42.6%)	93 (46.7%)		84 (42.0%)	42 (42.0%)	42 (42.0%)	
≥5cm	164 (54.7%)	58 (57.4%)	106 (53.3%)		116 (58.0%)	58 (58.0%)	58 (58.0%)	
MVI				*0.090*				*1.000*
-	238 (79.3%)	74 (73.3%)	164 (82.4%)		146 (73.0%)	73 (73.0%)	73 (73.0%)	
+	62 (20.7%)	27 (26.7%)	35 (17.6%)		54 (27.0%)	27 (27.0%)	27 (27.0%)	
PVTT				*0.453*				*0.833*
-	247 (82.3%)	86 (85.1%)	161 (80.9%)		174 (87.0%)	86 (86.0%)	88 (88.0%)	
+	53 (17.7%)	15 (14.9%)	38 (19.1%)		26 (13.0%)	14 (14.0%)	12 (12.0%)	

#### 3.1.1 Effect of postoperative adjuvant therapy on OS in HCC patients with high-risk recurrence factors

**Figure 2:**
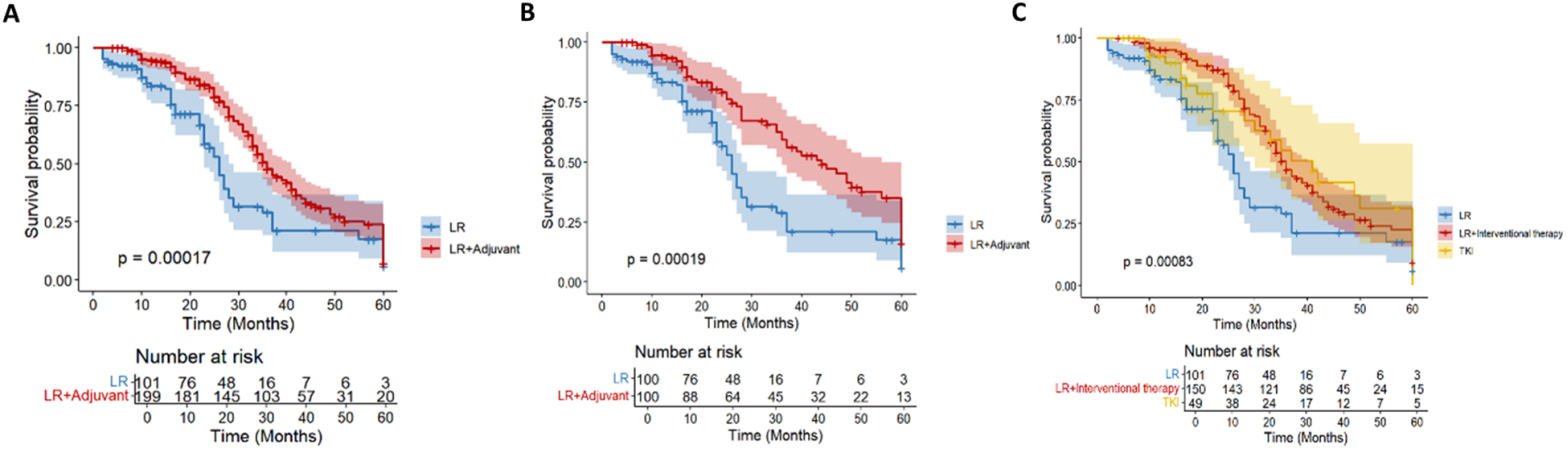
Kaplan–Meier analysis of OS in the entire cohort **(A)** and in the propensity score-matched cohort **(B)** of HCC patients with high-risk recurrence after liver resection. Kaplan–Meier analysis of different adjuvant therapy of OS in the entire cohort **(C)** of HCC patients with high-risk recurrence after liver resection.

During the follow-up period, 178 (59.3%) patients died in the entire cohort. Among the dead patients, 53 patients (52.5%) were in the LR group and 125 patients (62.8%) were in the LR+ adjuvant therapy group. In the whole cohort, the 1-year, 3-year and 5-year OS rates of the LR+ adjuvant therapy group were 94.7%, 47.8% and 7.2%, respectively, and those of the LR group were 83.5%, 29.0% and 5.9%, respectively (**Figure 2A**). After propensity matching, the 1-year, 3-year, and 5-year OS rates of the LR+ adjuvant therapy group were 82.2%, 46.0%, and 20.2%, respectively, and those of the LR group were 63.4%, 33.1%, and 17.2%, respectively (**Figure 2B**). In the whole cohort and the matched cohort, the OS of the LR+ adjuvant therapy group was significantly better than that of the LR group (HR, 0.55; 95% CI: 0.39-0.76; p< 0.001); (HR, 0.47; 95% CI: 0.32-0.71; p< 0.001). Univariate COX regression analysis was performed in the matching cohort, and AFP, ALT, AST, ALB, tumor diameter, PVTT and TACE were statistically significant. Univariate analysis with statistical significance was included in multivariate Cox regression analysis. Multivariate results showed that AFP (HR, 1.818; 95% CI: 1.054-3.316; p= 0.032), ALB (HR, 0.953; 95% CI: 0.910-0.998; p= 0.041), adjuvant therapy (HR, 0.477; 95% CI: 0.306-0.745; p= 0.001), tumor diameter (HR, 2.389; 95% CI: 1.078-5.607; p= 0.032), PVTT (HR, 2.765; 95% CI: 1.303-5.297; p= 0.008) and TACE (HR, 0.587; 95% CI: 0.372-0.927; p= 0.022) was a significant factor associated with OS in HCC patients with high-risk recurrence factors (**Table 2**).

**Table 2:** Univariate and multivariate Cox-regression analyzes predicting overall survival in the PSM cohort.

Characteristics	Univariate Cox-regression analyzes		Multivariate Cox-regression analyzes	
	Hazard ratio (95% CI)	*P*	Hazard ratio (95%CI)	*P*
Age	1.096 (0.733, 1.637)	0.655		
Sex	0.898 (0.574, 1.402)	0.635		
Hepatitis	0.964 (0.602, 1.543)	0.879		
Cirrhosis	1.155 (0.779, 1.113)	0.473		
AFP	**1.573 (1.017, 2.435)**	**0.042**	**1.818 (1.054, 3.136)**	**0.032**
ALT	**1.003 (1.000, 1.006)**	**0.024**	0.997 (0.989, 1.004)	0.384
AST	**1.003 (1.001, 1.006)**	**0.004**	1.004 (0.997, 1.011)	0.220
ALB	**0.943 (0.907, 0.980)**	**0.003**	**0.953 (0.910, 0.998)**	**0.041**
TBIL	1.011 (0.998, 1.025)	0.101		
Bleed	1.000 (1.000, 1.001)	0.017		
Blood transfusion	1.236 (0.787, 1.941)	0.358		
High risk				
Tumor number	0.639 (0.386, 1.057)	0.081		
Tumor diameter	**1.570 (1.084, 2.354)**	**0.029**	**2.389 (1.078, 5.607)**	**0.032**
MVI	0.876 (0.550, 1.394)	0.575		
PVTT	**1.772 (1.016, 3.092)**	**0.044**	**2.765 (1.303, 5.297)**	**0.008**
Treatment	**0.498 (0.328, 0.757)**	**0.001**	**0.477 (0.306, 0.745)**	**0.001**
TACE	**0.593 (0.396, 0.889)**	**0.011**	**0.587 (0.372, 0.927)**	**0.022**
HAIC	0.452 (0.143,1.429)	0.176	0.205 (0.061, 0.694)	0.011
TKI	0.767 (0.410, 1.433)	0.405		

p < 0.05.

In the overall cohort (n= 300), according to the postoperative adjuvant treatment, the patients were divided into LR group (n= 101), intervention group (n= 150) and TKI group (n= 49). There were significant differences in OS among the three groups (p< 0.001; **Figure 2C**). The survival rate of the intervention group was significantly better than that of the other two groups. There were still 15 cases survived at 60 months of follow-up, which significantly improved OS (HR, 0.54; 95% CI: 0.38-0.76). Although the TKI group intersected with the middle stage of the LR group, it still showed a good survival advantage (HR, 0.58; 95% CI: 0.35-0.94).

#### 3.1.2 Effect of postoperative adjuvant therapy on DFS in HCC patients with high-risk recurrence factors

**Figure 3:**
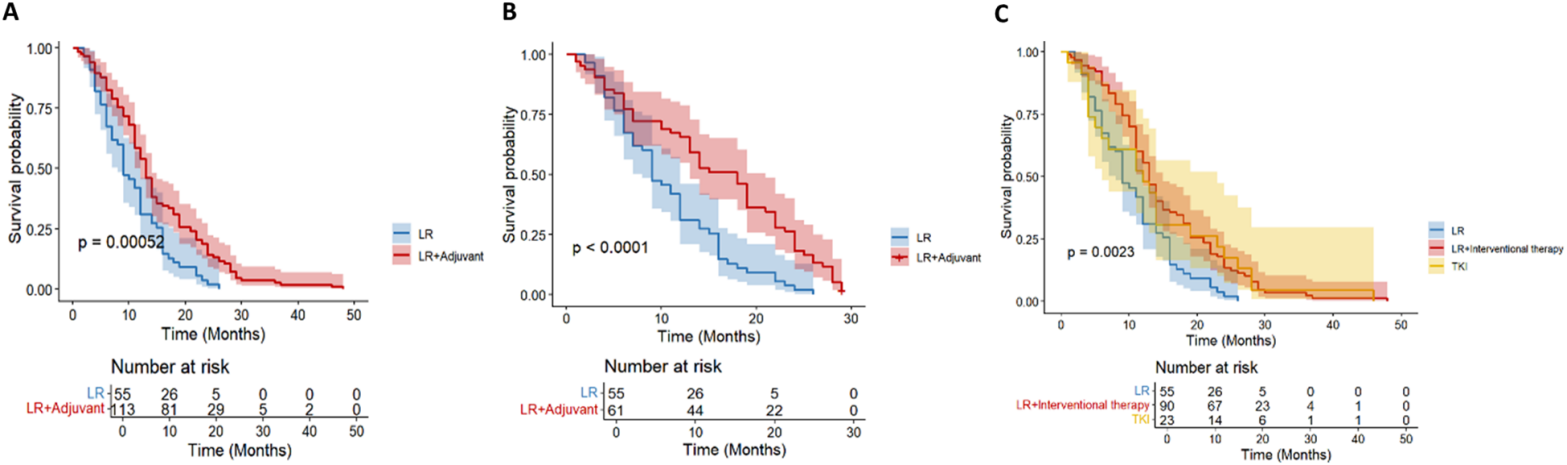
Kaplan–Meier analysis of DFS in the entire cohort **(A)** and in the propensity score-matched cohort **(B)** of HCC patients with high-risk recurrence after liver resection. Kaplan–Meier analysis of different adjuvant therapy of DFS in the entire cohort **(C)** of HCC patients with high-risk recurrence after liver resection.

A total of 168 patients (56%) had tumor recurrence during the follow-up period. In the entire cohort, 55 patients (54.5%) in the LR group had tumor recurrence, and 113 patients (56.8%) in the LR+ adjuvant therapy group had tumor recurrence. Before propensity matching, the 1-year, 3-year, and 5-year DFS rates of the LR+ adjuvant therapy group were 54%, 2.7%, and 0%, respectively, and those of the LR group were 30.9%, 0%, and 0%, respectively (**Figure 3A**). After propensity matching, the 1-year, 3-year and 5-year DFS rates of LR+ adjuvant therapy group were 53.4%, 2.6% and 0%, respectively, and those of LR group were 30.9%, 0% and 0%, respectively (**Figure 3B**). The DFS of the LR+ adjuvant therapy group was significantly better than that of the LR group (HR, 0.55; 95% CI: 0.40-0.77; p = 0.001); (HR, 0.43; 95% CI: 0.29-0.65; p< 0.001). Univariate COX regression analysis was performed in the matching cohort, and AFP, multiple tumors, PVTT, MVI and TACE were statistically significant. Univariate analysis with statistical significance was included in multivariate Cox regression analysis. Multivariate results showed that AFP (HR, 0.330; 95% CI:0.160-0.683; p= 0.003), adjuvant therapy (HR, 0.394; 95% CI: 0.253-0.614; p< 0.001), multiple tumors (HR, 2.011; 95% CI: 1.005-4.022; p = 0.048), MVI (HR, 4.129; 95% CI: 2.008-8.489; p < 0.001), PVTT (HR, 2.436; 95%CI: 1.287-4.611; p= 0.006) and TACE (HR, 0.359; 95% CI: 0.226-0.571; p< 0.001) was a significant factor associated with DFS in HCC patients with high-risk recurrence factors (**Table 3**).

**Table 3:** Univariate and multivariate Cox-regression analyzes predicting disease-free survival in the PSM cohort.

Characteristics	Univariate Cox-regression analyzes		Multivariate Cox-regression analyzes	
	Hazard ratio (95% CI)	*P*	Hazard ratio (95% CI)	*P*
Age	1.040 (0.714, 1.517)	0.837		
Sex	0.738 (0.480, 1.134)	0.165		
Hepatitis	0.724 (0.479, 1.093)	0.124		
Cirrhosis	0.891 (0.617, 1.286)	0.538		
AFP	**1.641 (1.047, 2.573)**	**0.031**	**0.330 (0.160, 0.683)**	**0.003**
ALT	1.002 (0.999, 1.005)	0.299		
AST	1.000 (0.997, 1.003)	0.944		
ALB	0.998 (0.960, 1.037)	0.919		
TBIL	1.007 (0.991, 1.022)	0.398		
Bleed	1.000 (1.000, 1.001)	0.506		
Blood transfusion	1.231 (0.793, 1.909)	0.354		
High risk				
Tumor number	**0.631 (0.400, 0.996)**	**0.048**	**2.011 (1.005, 4.022)**	**0.048**
Tumor diameter	1.288 (0.884, 1.877)	0.188	**3.018 (1.540, 5.912)**	**0.001**
MVI	**1.523 (1.000, 2.323)**	**0.050**	**4.129 (2.008, 8.489)**	**<0.001**
PVTT	**1.691 (1.011, 2.827)**	**0.045**	**2.436 (1.287, 4.611)**	**0.006**
Treatment	**0.456 (0.307, 0.678)**	**<0.001**	**0.394 (0.253, 0.614)**	**<0.001**
TACE	**0.469 (0.317, 0.695)**	**<0.001**	**0.359 (0.226, 0.571)**	**<0.001**
HAIC	0.668 (0.272, 1.643)	0.380		
TKI	0.931 (0529, 1.641)	0.806		

p < 0.05.

Postoperative adjuvant therapy was further divided into hepatectomy group (n= 101), intervention group (n= 150) and TKI group (n= 49) according to the postoperative adjuvant therapy. There were significant differences in DFS among the three groups (p< 0.001; **Figure 3C**). Intervention group (HR, 0.55; 95% CI: 0.39-0.77) and TKI group (HR, 0.58; 95% CI: 0.35-0.96) DFS were significantly improved.

#### 3.1.3 Stratified analysis of high-risk recurrence factors

We further stratified analysis based on different high- risk recurrence factors, aiming to explore the differences in the efficacy of postoperative adjuvant therapy in specific subgroups, so as to provide more accurate treatment guidance for clinical practice. The following factors were analyzed in detail, including preoperative AFP level, tumor diameter, multiple tumors, MVI and PVTT.

### 3.2 High AFP level

**Figure 4:**
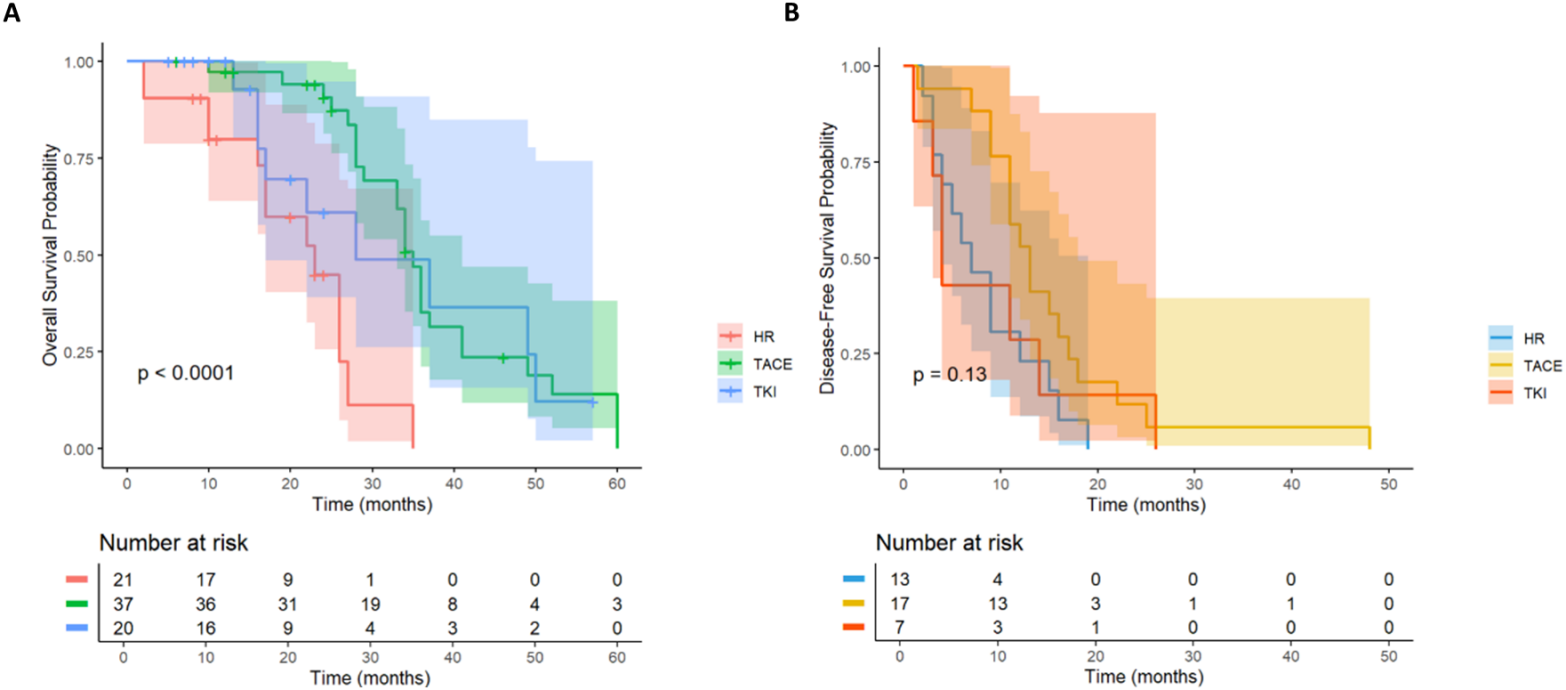
Kaplan-Meier analysis of different adjuvant therapy of OS **(A)** and DFS **(B)** in high-risk recurrent HCC patients after liver resection with AFP ≥400ng/mL.

For HCC patients with high Alpha-fetoprotein (AFP) level (≥ 400ng/mL) recurrence risk, according to the survival analysis of different adjuvant treatment regimens, there was a significant difference in OS between the LR group, the TACE treatment group and the TKI treatment group (**Figure 4A**), while the difference in DFS was not significant (**Figure 4B**). In terms of OS, the TACE group significantly prolonged the survival of patients compared with the LR group (HR = 0.18, 95% CI = 0.08-0.4), and the TKI group also showed significant improvement (HR = 0.24, 95% CI = 0.29-0.61). Although the overall DFS improvement was not statistically significant, the TACE therapy group (HR = 0.48, 95% CI = 0.23-1.01) showed a clinically significant reduction in the risk of recurrence compared with the TKI group (HR = 0.79, 95% CI = 0.3-2.05). The above results prove that for patients with high AFP levels, regardless of the DFS results, the application of postoperative adjuvant therapy can improve the OS of patients.

### 3.3 Tumor diameter ≥ 5cm

**Figure 5:**
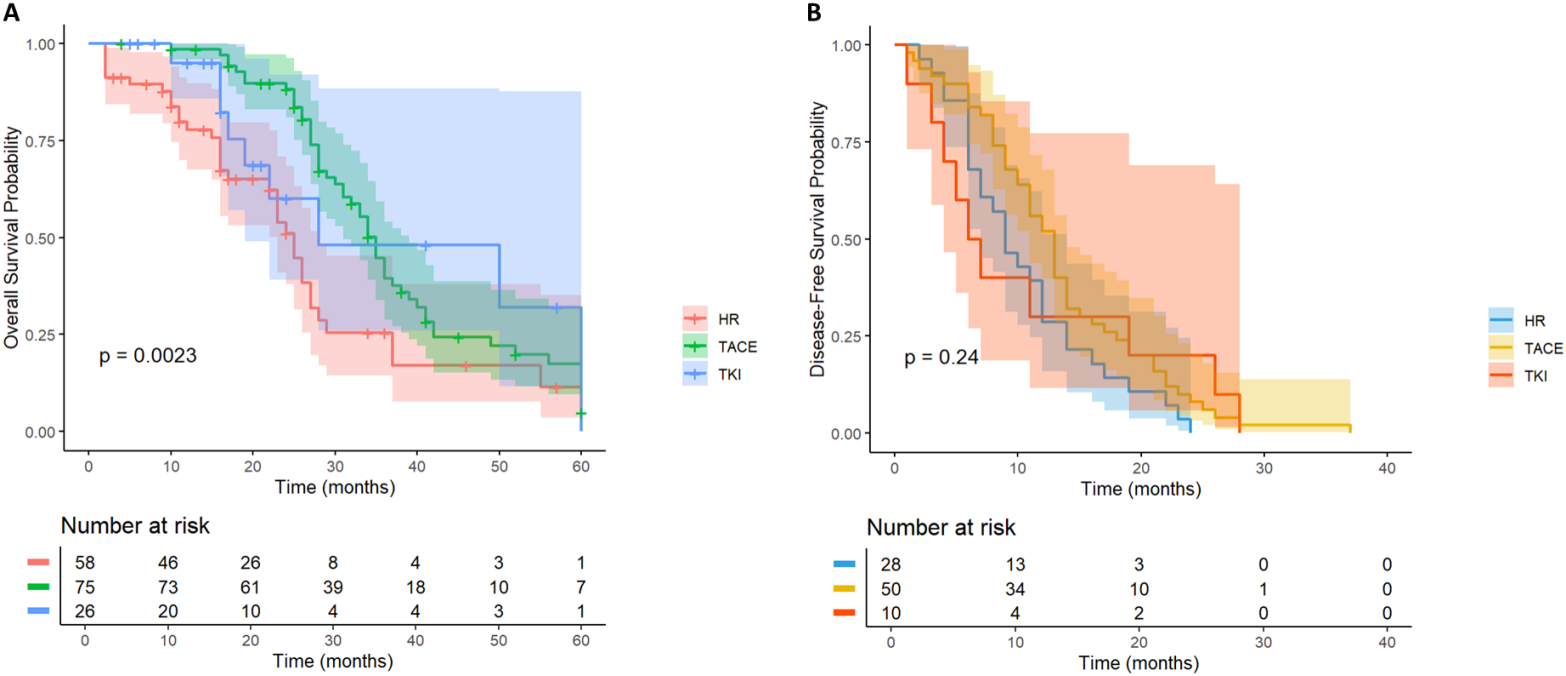
Kaplan-Meier analysis of OS **(A)** and DFS **(B)** in high-risk recurrent HCC patients after liver resection with tumor diameter≥5cm.

In HCC patients with high-risk recurrence factors of tumor diameter≥ 5cm (n= 164), they were divided into TACE group (n= 80), TKI group (n= 26) and LR group (n= 58) according to treatment methods. There was a significant difference in OS (**Figure 5A**) among the three groups (P = 0.0012). TACE group (HR = 0.46; the survival probability of 95% CI = 0.30-0.70) was significantly better than the other two groups. The long-term survival of the whole population in the TKI group was not significantly improved (HR = 0.51; 95% CI = 0.24-1.06).

In the DFS analysis (**Figure 5B**), the DFS of the TACE group was significantly better than that of the LR group (HR = 0.62; 95% CI = 0.39-0.99), while the DFS of the whole population in the TKI group was not significantly improved (HR = 0.73; 95% CI = 0.35-1.54). For HCC patients with tumor diameter≥ 5cm, postoperative TACE therapy can significantly improve OS and DFS, while TKI treatment is not effective.

**Figure 6:**
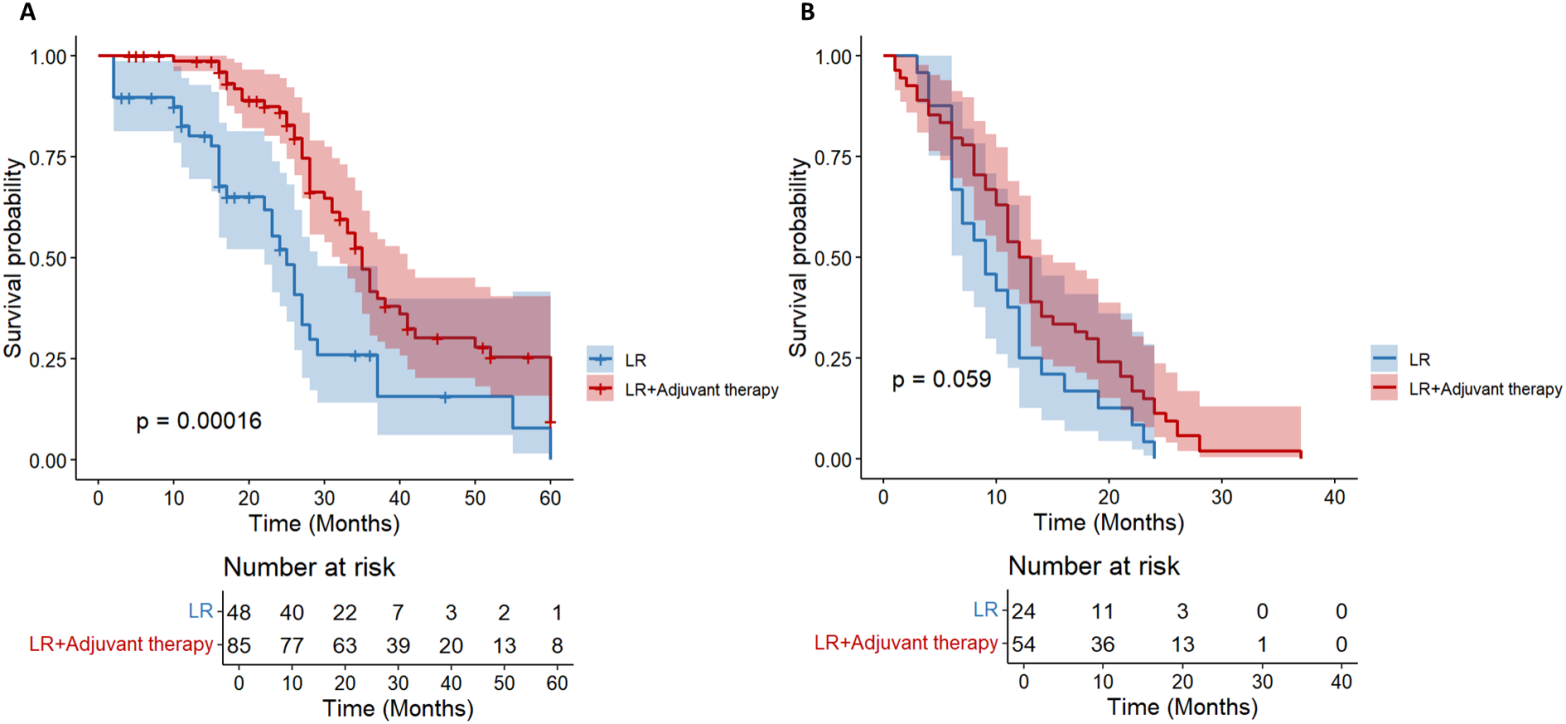
Kaplan-Meier analysis of OS **(A)** and DFS **(B)** in high-risk recurrent HCC patients after liver resection with tumor diameter 5–10 cm.

We further stratified patients with a diameter of ≥ 5cm into two groups of 5-10cm and > 10cm. In the cohort of HCC patients with a tumor diameter of 5–10 cm and no other high-risk recurrence factors, the postoperative adjuvant treatment group showed a clinically significant survival advantage. It is worth noting that the benefit of OS is particularly prominent (HR = 0.42; 95%CI: 0.26-0.66, p< 0.01, **Figure 6A**). Compared with the significant improvement of OS, the benefit of DFS was relatively limited but still had certain clinical significance (HR = 0.62; 95% CI: 0.38-1.01, p= 0.059, **Figure 6B**).

**Figure 7:**
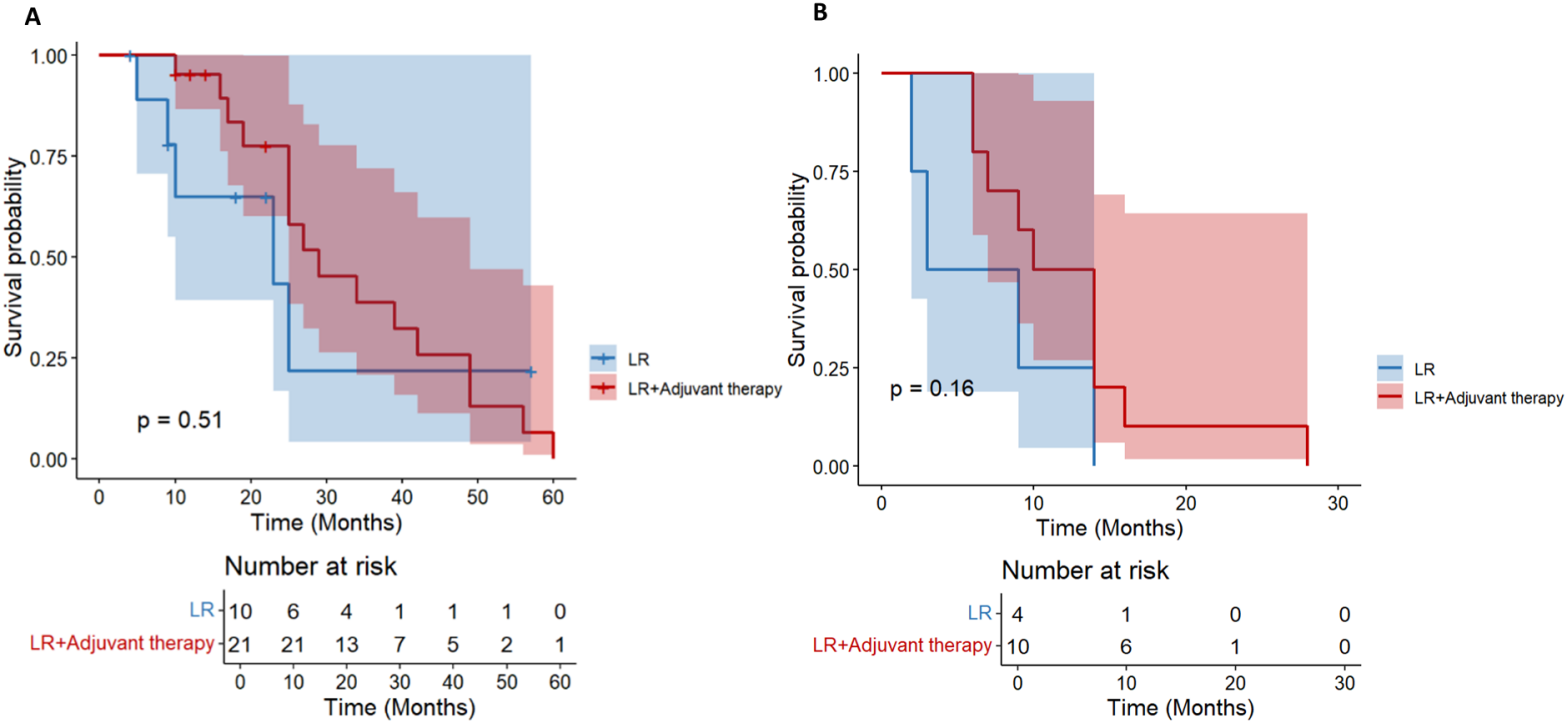
Kaplan-Meier analysis of OS **(A)** and DFS **(B)** in high-risk recurrent HCC patients after liver resection with tumor diameter > 10 cm.

For HCC patients with tumor diameter> 10 cm, there was no significant difference in OS between the LR group and the postoperative adjuvant therapy group (HR = 0.71; 95% CI: 0.25-1.99, p= 0.51, **Figure 7A**). The initial sample size of the LR group was small (n= 10), and no survival patients were followed up to 60 months. The initial sample size of the postoperative adjuvant treatment group was 21 cases, but only 1 case survived at 60 months, indicating that the overall prognosis of patients with tumor diameter> 10 cm was extremely poor. There was no significant difference in DFS between the two groups (HR = 0.43; 95% CI: 0.13-1.44, p= 0.16, **Figure 7B**), and all showed a rapid downward trend. All patients had recurrence at 30 months. Overall, in HCC patients with tumor diameter> 10 cm, postoperative adjuvant therapy did not bring significant OS or DFS benefits compared with simple hepatectomy.

### 3.4 Multiple tumors

**Figure 8:**
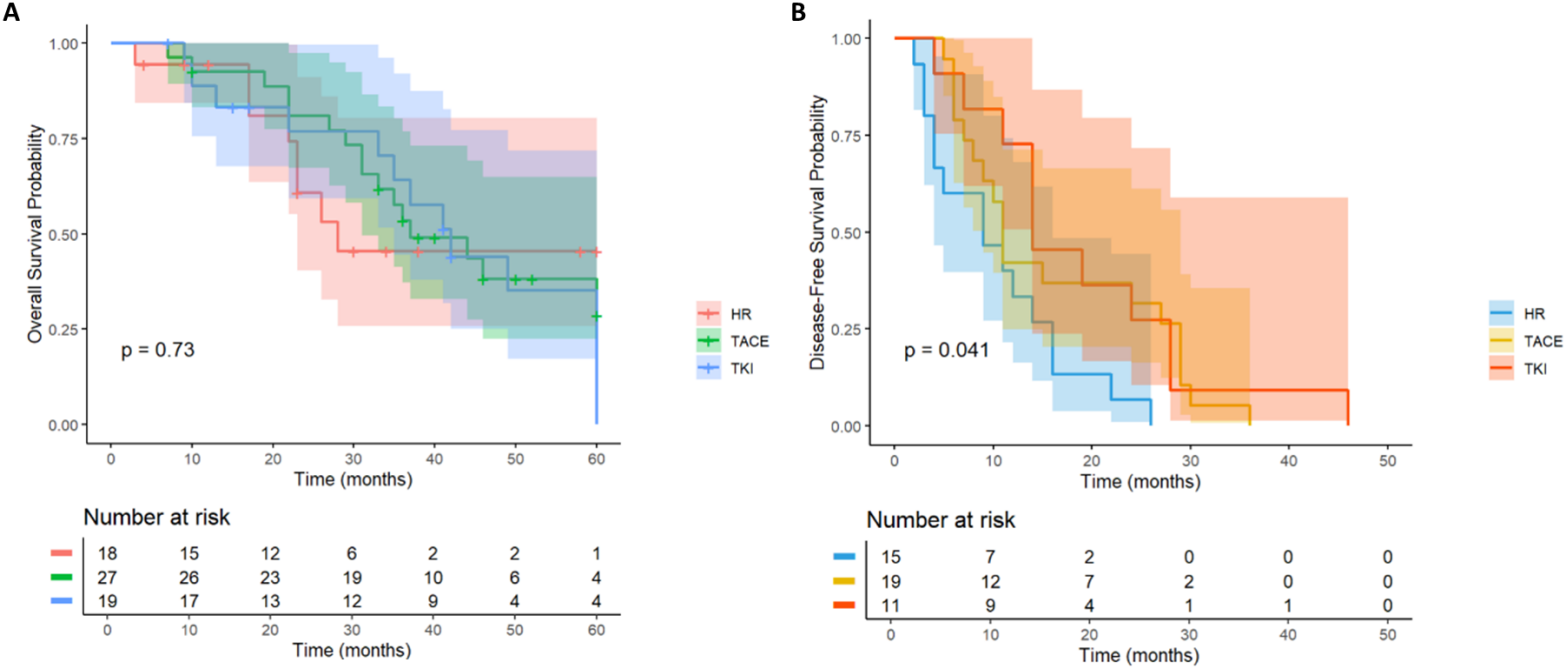
Kaplan–Meier analysis of OS and DFS **(A, B)** of HCC patients with multinodular tumor after liver resection.

A total of 66 HCC patients with high risk of multiple tumor recurrence who underwent radical hepatectomy were included. According to the postoperative adjuvant therapy, they were divided into three groups: LR group (n= 18), TACE group (n= 29) and TKI group (n= 19). Different adjuvant therapies had a significant effect on DFS (P = 0.041, **Figure 8B**). Further comparison between groups showed that the median DFS of the TACE group was 28.6 months, which was significantly better than 16.2 months of the LR group (HR = 0.46; 95% CI: 0.22-0.97), and the median DFS in the TKI group was 24.3 months, which also showed a statistical advantage compared with the LR group (HR = 0.4; 95% CI: 0.18-0.93). It is worth noting that although the DFS of the TACE group and the TKI group was significantly prolonged, there was a significant difference between the two treatment groups, suggesting that TKI treatment had a better clinical effect in delaying tumor recurrence (HR = 2.15; 95% CI: 1.03-4.47). In the OS analysis (**Figure 8A**), the TACE group (HR = 0.74; 95% CI: 0.32-1.73) and TKI group (HR = 1; 95% CI: 0.41-2.42) were not observed significant statistical differences. Postoperative adjuvant therapy can delay recurrence, but failed to transform into a survival advantage. In DFS, the TKI group performed better than the TACE group and can be used as the first choice for patients with multiple tumors. For OS, we need to further expand the sample size to extend the follow-up time to clarify the mechanism of OS improvement.

### 3.5 MVI

**Figure 9:**
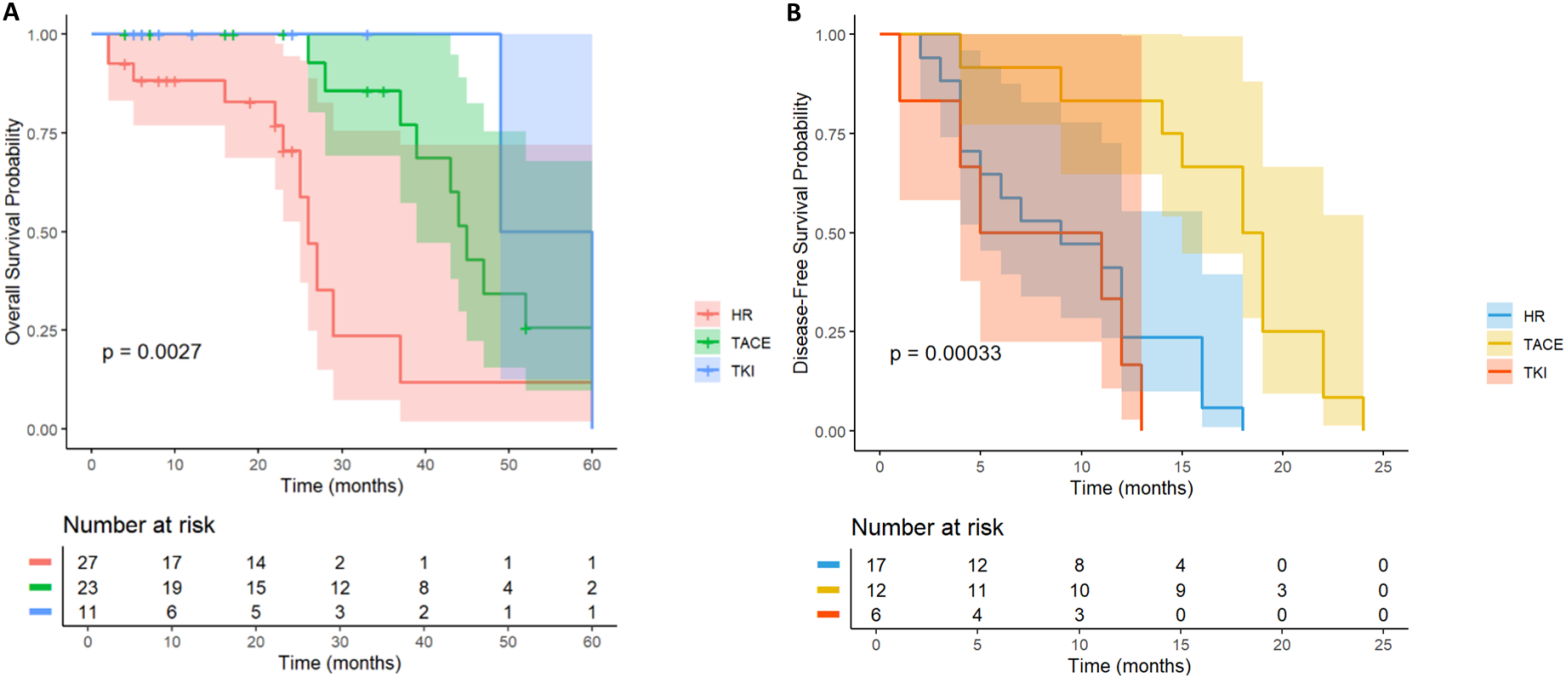
Kaplan-Meier analysis of OS **(A)** and DFS **(B)** in high-risk recurrent HCC patients after liver resection with MVI.

In MVI-positive HCC patients (n= 62), the OS of TACE therapy group (n= 24) and TKI treatment group (n= 11) was significantly better than that of LR group (n= 27) (p< 0.001, **Figure 9A**). Patients with postoperative TACE therapy (HR = 0.32, 95% CI: 0.24-0.77) or TKI therapy (HR = 0.19, 95% CI: 0.04-0.86) had significantly longer OS. In terms of DFS, postoperative TACE therapy significantly reduced the risk of recurrence (HR = 0.17, 95% CI: 0.06-0.46), while TKI treatment did not show a significant effect on delaying recurrence (HR = 1.53, 95%CI: 0.58-4.00, **Figure 9B**). Further observation showed that 3 patients in the TACE group had no recurrence at 20 months after operation, while patients in the LR group and the TKI group had recurrence or death within 15 months after operation. For MVI-positive HCC patients, postoperative TACE therapy has shown significant advantages in prolonging survival and reducing the risk of recurrence.

### 3.6 PVTT

**Figure 10:**
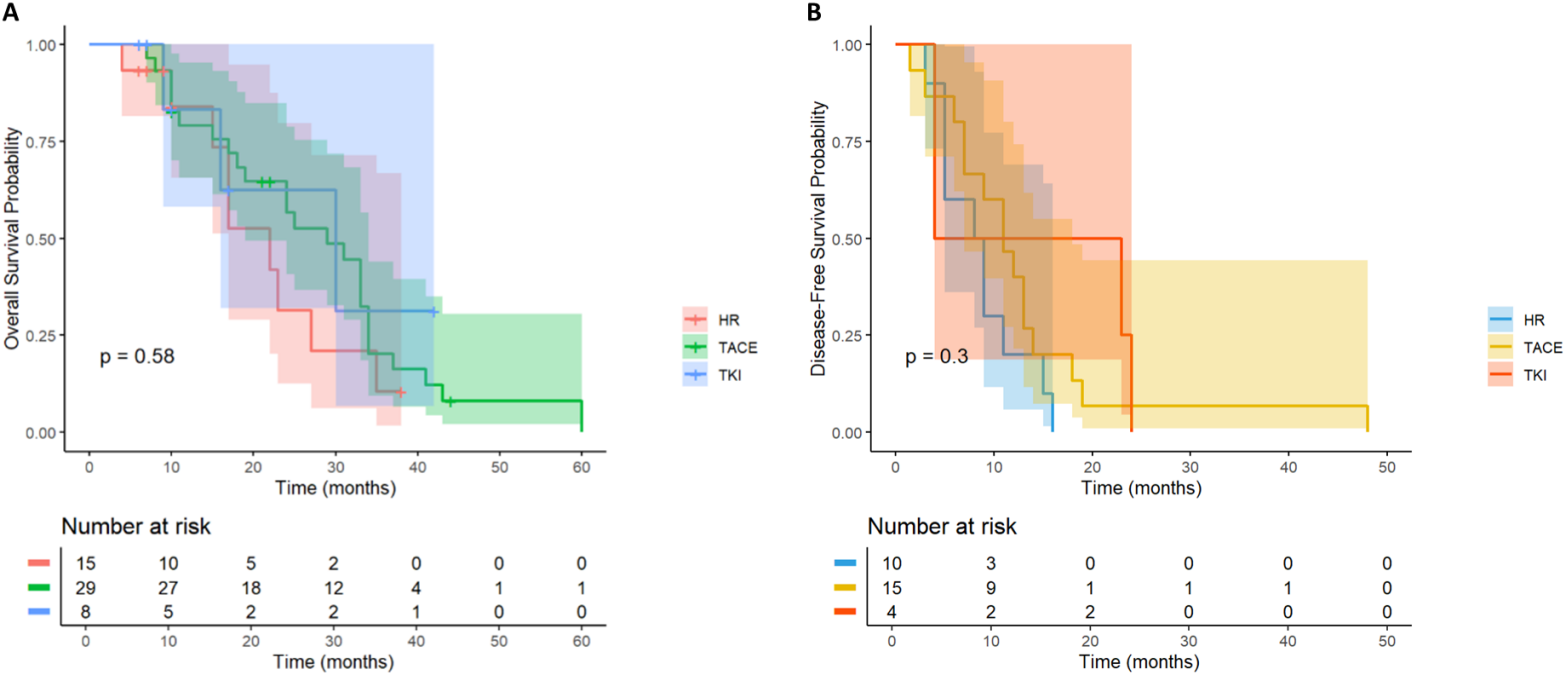
Kaplan-Meier analysis of OS **(A)** and DFS **(B)** in high-risk recurrent HCC patients after liver resection with PVTT.

In HCC patients with PVTT, there was no significant difference in the OS curve between the LR group (n = 15) and the TACE therapy group (n= 30) and the TKI group (n= 8) (p= 0.51, **Figure 10A**). All patients in the LR group died within 40 months after operation, and all patients in the TKI treatment group died within 50 months after operation, while 1 patient (3.3%) in the TACE treatment group survived at 60 months after operation. However, the median survival time of the three groups was similar, suggesting that postoperative TACE therapy (HR = 0.66, 95% CI: 0.30-1.44) or TKI targeted therapy (HR = 0.58, 95% CI: 0.16-2.17) did not significantly improve the long-term survival prognosis of patients with PVTT. Further analysis of the DFS revealed (p= 0.27, **Figure 10B**) that there was no significant difference in the recurrence rate between the LR group and the TACE group (HR = 0.57; 95% CI: 0.25-1.31) and the TKI group (HR = 0.4; 95% CI: 0.11-1.42). No statistically significant differences were observed.

## 4 Discussion

**Figure 11:**
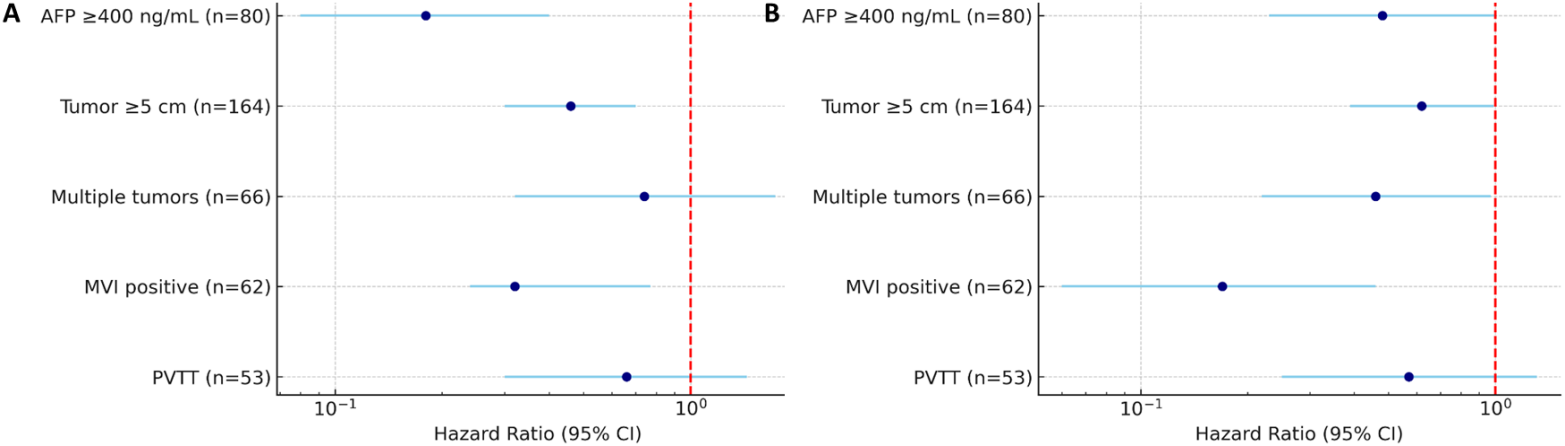
Forest plot summarizing HR for OS **(A)** and DFS **(B)** in HCC patients with high-risk factors. Subgroups include patients with AFP≥400 ng/mL (n=80), tumor diameter≥5 cm (n=164), multiple tumors (n=66), MVI (n=62), and PVTT (n=53). HRs are shown with 95% CI.

This study systematically evaluated the effects of different postoperative adjuvant treatment strategies on the survival outcomes of HCC patients with high-risk recurrence factors (MVI, PVTT, giant tumors, multiple tumors, etc.). We did not establish a separate group for HAIC due to its very small sample size (n=9), which would have led to unreliable statistical comparisons. Therefore, the primary analysis focused on TACE and TKI. The results showed that postoperative adjuvant therapy can significantly improve OS and DFS in patients with high-risk recurrence factors, and TACE therapy showed the most clear clinical benefits. The results of this study showed that the survival advantage of TACE therapy was particularly prominent in patients with high AFP level or tumor diameter≥ 5 cm compared with radical hepatectomy alone. In addition, TACE therapy significantly reduced the risk of recurrence in MVI-positive patients, while TKI treatment also significantly reduced recurrence in patients with high AFP levels or multiple tumors, further consolidating the position of these two adjuvant therapies in postoperative adjuvant therapy for high-risk patients (**Figure 11**).

With the advancement of surgical techniques and the optimization of perioperative management, the incidence of surgery-related mortality and complications in HCC patients has decreased significantly (2, 16). However, surgical resection alone is difficult to completely remove potential micrometastases or residual tumor cells, and high postoperative recurrence rate is still one of the main obstacles affecting long-term efficacy. This challenge has prompted clinicians to continuously explore more effective postoperative adjuvant treatment strategies to delay recurrence and prolong survival. Postoperative adjuvant therapy can control the residual tumor burden locally or systematically, and plays an important role in the early postoperative stage. However, for patients with high-risk recurrence factors, it is still controversial which adjuvant therapy should be selected after surgery.

At present, most of the research on postoperative TACE therapy focuses on HCC patients with early or no obvious high-risk recurrence factors, and the research evidence for high-risk recurrence factors is still scarce (17, 18). In the existing studies, there are still great differences in the efficacy of postoperative TACE therapy in HCC patients with high-risk recurrence. On the one hand, some studies have pointed out that postoperative TACE therapy has failed to significantly improve survival outcomes (19). On the other hand, some studies have reported that postoperative TACE can effectively prolong DFS and OS, suggesting that it may have a positive effect in a specific population (20). The results of this study showed that postoperative TACE therapy significantly improved OS in patients with high-risk recurrence factors, and was established as an independent protective factor for OS and DFS in multivariate analysis. Consistent with this, in the latest Meta-analysis of 12 randomized controlled trials, it was also pointed out that postoperative TACE can reduce the risk of recurrence of HCC patients with high-risk recurrence factors by about 30%, further verifying the clinical value of postoperative TACE adjuvant therapy (20).

In terms of TKI treatment, in terms of overall high-risk recurrence factors, our study found that TKI monotherapy significantly improved OS and DFS, but this is inconsistent with the conclusion of the STORM study (21), which failed to show significant efficacy of TKI monotherapy in HCC patients. Specifically, the conclusions of the STORM study may be limited by various factors such as study design, patient selection criteria, and the type of TKI drugs used, and did not specifically analyze HCC patients with high-risk recurrence factors, while our study focused on specific high-risk recurrence factors. TKI treatment can effectively improve OS in patients with high levels of AFP, and can effectively improve DFS in patients with multiple tumors. These results suggest that the benefit of adjuvant TKI therapy may be more pronounced in biologically aggressive tumors or patients at higher risk of recurrence.

Further stratified analysis of high-risk recurrence factors showed that patients with high AFP levels benefited significantly from postoperative TACE therapy, suggesting that high AFP levels are not only a marker of poor prognosis of HCC, but also a marker of priority for TACE therapy. Postoperative TACE treatment, by embolizing the blood supply artery of the lesion, not only directly reduces the nutritional supply of residual tumor cells, but also induces the “normalization” effect of blood vessels, stabilizes the tumor microenvironment, and inhibits the formation of micrometastases (22–24). HAIC treatment, especially the 5-FU component, has the effect of selectively removing Treg cells, thereby reshaping the local immune microenvironment to a certain extent, relieving immunosuppression and restoring anti-tumor immune activity (25). This series of mechanisms provide a reasonable biological basis for patients with high AFP levels to obtain greater survival benefits from TACE therapy. Although in this study, the DFS improvement of the high AFP level subgroup was not statistically significant, its OS showed significant benefits, suggesting that TACE therapy can significantly prolong the survival time of these patients. This separation of DFS and OS results may reflect the potential differences in TACE therapy in delaying tumor progression and controlling extrahepatic distant metastasis. As one of the pro-angiogenic factors, AFP can promote the abnormal proliferation of blood vessels by binding to vascular endothelial growth factor receptor 2 (VEGFR2), increase the permeability and structural instability of tumor blood vessels, and accelerate the process of hematogenous metastasis (26). AFP also promotes B-cell lymphoma-2 (Bcl-2) gene expression through the Retinoic acid and retinoid acid receptor (RA-RAR) signaling pathway, accelerating the progression of HCC (27). OS was significantly improved in patients with high AFP levels after TKI treatment, which was consistent with the results of the REFLECT study (13). In biology, high AFP level is closely related to the activation of Fibroblast growth factor receptor (FGFR) pathway (28), and TKI drugs can effectively block FGFR and VEGFR signaling pathways, thereby inhibiting tumor angiogenesis, improving tumor perfusion, and enhancing follow-up treatment response (29–31). TACE therapy can control tumor growth and metastasis more directly and locally by embolization of tumor feeding arteries and remodeling of immune microenvironment. In contrast, although TKI has advantages in inhibiting angiogenesis, its therapeutic mechanism is more focused on systemic therapy, which may be less intuitive and effective than TACE therapy for the adjustment of local microenvironment. In summary, TACE therapy may be a more preferred choice in patients with high AFP, because they can provide more comprehensive biological TACE and prolong the survival of patients through direct local therapeutic effects, vascular “normalization” effects, and immune remodeling mechanisms (32, 33). TKI treatment has its unique advantages in inhibiting tumor angiogenesis and systemic treatment, and is suitable for combined use with TACE therapy, especially in patients with extrahepatic metastasis (34).

In patients with tumor diameter≥ 5 cm, postoperative TACE therapy has brought significant benefits in both DFS and OS. Giant tumors not only indicate higher tumor load, but also are accompanied by strong angiogenesis and hypoxia environment, which activates HIF-1α, VEGF and other pathways to promote tumor cell invasion, metastasis and immunosuppression (35, 36). For patients with tumor diameter≥ 5 cm but≤ 10 cm, postoperative TACE therapy can accurately remove occult micrometastases. Studies have confirmed that 92.3% of micrometastases with a diameter of< 1 cm rely on hepatic artery blood supply, while TACE can achieve selective killing through local embolization and high concentration of chemotherapeutic drugs (hepatic artery administration concentration is 9 times higher than intravenous administration), so that the clearance rate of microvascular tumor thrombus is increased to 73.6% (LR group 34.2%, p< 0.001) (37); TACE therapy can inhibit the VEGF signal at the peak of postoperative angiogenesis (decreased by 72.3%), and transiently activate anti-tumor immunity. For example, the infiltration of CD8 + T cells increased by 3.5 times (38, 39). These mechanisms together explain the fact that Chen et al. (40) reported that the 1-year and 2-year DFS of patients with HCC≥ 5 cm who received postoperative TACE therapy was significantly better than that of the LR group. Li et al. (41) reported that postoperative TACE can significantly improve the survival of patients with liver cancer with a diameter of≥ 5 cm, but the efficacy is significantly reduced in patients with a diameter of> 10 cm. Kim et al. also observed that the objective response rate of TACE for tumors> 10 cm was significantly lower than that of small and medium-sized tumors (42). Although its mechanism may be related to factors such as hypoxia and immunosuppressive microenvironment that are more likely to occur in large-volume tumors (43, 44), in the postoperative context, > 10 cm tumors are more representative of high invasiveness and high risk of recurrence. Previous studies have shown that giant tumors are often accompanied by a higher proportion of MVI, satellite nodules and incomplete envelopes, suggesting a stronger metastatic tendency and worse biological behavior, which significantly affects postoperative disease-free survival and overall survival. Therefore, HCC> 10 cm should be considered as a high-risk recurrence subgroup after surgery, and more active adjuvant therapy TACE and follow-up management are needed. Nevertheless, the relatively small sample size of patients with tumors >10 cm in the LR group may have limited the statistical power of our analysis; thus, the findings in this subgroup should be interpreted with caution.

Multiple tumors usually reflect the spread or multifocal origin of intrahepatic tumors, which are usually accompanied by a higher risk of recurrence and poor treatment response. In this study, although different adjuvant treatment strategies did not show statistical differences in OS, the effect of TKI treatment group was slightly better than that of TACE treatment group in terms of DFS, suggesting that TKI drugs may show stronger ability to control recurrence in patients with multiple tumors by inhibiting the growth of neovascularization and latent metastases (45). Studies have shown that tumor tissues of patients with multiple liver cancers are often accompanied by higher VEGF expression levels and more active angiogenesis pathways (46). TKI drugs such as sorafenib and lenvatinib can effectively reduce the risk of tumor recurrence through multi-target anti-angiogenesis. Li et al. reported in RCT (Randomized Controlled Trial) that postoperative TACE can improve survival outcomes in patients with multiple tumors (41). However, due to the large heterogeneity between tumors and the complex recurrence path, it is difficult for adjuvant therapy to completely remove all potential residual lesions. Pathological studies have shown that multiple HCC usually has a polyclonal origin and a high gene mutation load, making it easier to evade immune surveillance. In addition, multiple tumors also increase the difficulty of local treatment TACE and increase the possibility of recurrence. Therefore, for patients with multiple tumors, individualized adjuvant therapy based on clinical stage and molecular characteristics may be the key to improve long-term prognosis.

MVI is one of the important risk factors for early recurrence of HCC after surgery, and its existence reflects the stronger invasiveness of the tumor and the potential tendency of hematogenous metastasis (41). In this study, after postoperative adjuvant therapy, TACE therapy significantly improved the OS and DFS of MVI-positive patients, while TKI treatment was beneficial to OS, but the improvement in DFS was not good. This result suggests that although postoperative adjuvant therapy can prolong survival, it may be difficult to completely overcome the high risk of recurrence caused by MVI. Existing studies have provided sufficient evidence for the efficacy of postoperative TACE therapy. A number of systematic reviews and meta-analyses agreed that TACE as a postoperative adjuvant therapy can significantly improve survival outcomes in MVI-positive HCC patients (47, 48). For example, a meta-analysis of 6977 patients in 24 studies conducted by Liang et al. showed that TACE could significantly prolong the OS and DFS of HCC patients with high recurrence risk factors (47). This result supports the important value of TACE as an adjuvant therapy in MVI-positive patients. In contrast, the application value of TKI therapy in these patients is still controversial. Although some studies have shown that TKI can prolong overall survival by inhibiting tumor angiogenesis and cell proliferation mechanisms (3, 49), there is still no consistent evidence on its control of postoperative recurrence. Especially in the context of highly invasive MVI, TKI treatment alone may be difficult to effectively curb the expansion and long-term recurrence of minimal residual lesions. In addition, the lack of reliable non-invasive prediction methods for MVI before surgery has led to the failure of some high-risk patients to obtain accurate adjuvant treatment interventions in time after surgery, which further affects their survival outcomes (50). This reality also highlights that the current treatment model still has significant deficiencies in MVI-positive patients. For MVI-positive HCC patients, a single mode of adjuvant therapy may be difficult to fully inhibit their recurrence potential. In the future, more attention should be paid to the exploration of individualized and multi-mode combined treatment strategies (51). While improving long-term survival, the recurrence rate should be reduced to provide a more effective treatment path for high-risk groups.

In HCC patients with PVTT, the results of our study showed that postoperative adjuvant therapy failed to significantly improve DFS and OS, suggesting that traditional postoperative adjuvant therapy had limited clinical benefits in such patients. PVTT is one of the most aggressive and high-risk manifestations of HCC, usually suggesting that the tumor has broken through the local limitations of the liver and entered the early stage of intrahepatic and extrahepatic metastasis (52). Even after radical resection, the prognosis of PVTT-positive patients is still very poor, which may be closely related to its own highly malignant biological characteristics, difficulty in completely removing tumor thrombus and potential residual lesions during operation, and limited postoperative treatment effect (53, 54). PVTT often causes obstruction of the main or primary branches of the portal vein, which in turn leads to intrahepatic blood flow remodeling and uneven perfusion. This abnormal hemodynamic environment promotes the re-dissemination of tumor cells and increases the risk of distant metastasis (55). At the same time, PVTT-related regions exhibit a significant immunosuppressive microenvironment, including regulatory T cell (Tregs) enrichment, antigen presentation disorders, and limited dendritic cell activation, which significantly weakens the anti-tumor immune response mediated by CD8^+^ T cells (56), resulting in limited postoperative immune-related adjuvant therapy. TACE mainly acts on the blood supply area of the hepatic artery, and about 30-50% of the blood supply in PVTT lesions depends on the portal vein system (57, 58), resulting in a serious shortage of drug concentration in the portal vein area, and the clearance rate of micrometastasis is less than 20%. In addition, portal hypertension caused by PVTT further limits the effective penetration of drugs in liver tissue (59), which weakens the local therapeutic effect of TACE and targeted drugs. The mutation frequency of key pathways such as FGFR4 in PVTT lesions is high, which has been proved to lead to a decrease in the efficacy of TKI drugs such as lenvatinib (60). PVTT-positive HCC patients are at a very high risk of recurrence and death after surgery, and traditional postoperative adjuvant therapy has little effect on its improvement. Future treatment strategies should explore the combination or sequential regimen of targeted therapy, immunotherapy and local therapy on the basis of precise classification, so as to break the dilemma of poor long-term prognosis of PVTT patients after operation.

With the evolving landscape of HCC management, there is increasing interest in postoperative combination strategies that integrate locoregional and systemic therapies. Recent studies suggest that combining TACE with TKI or immune checkpoint inhibitors (ICIs) may achieve synergistic effects by enhancing local tumor control, remodeling the tumor vasculature, and modulating the immune microenvironment, thereby reducing recurrence risk in high-risk populations (61–63). Similarly, HAIC combined with ICIs has shown promising efficacy in advanced HCC and may have potential in the adjuvant setting (64–66). Given the limited efficacy of monotherapy in certain subgroups—such as those with PVTT or tumor diameter≥5cm—future clinical trials should focus on rationally designed combination regimens to optimize postoperative outcomes (67).

It is worth noting that in the real-world retrospective cohort used in this study, once HCC patients relapse after surgery, their follow-up treatment is highly heterogeneous, including multiple strategies such as reoperation, interventional therapy, systemic therapy, and palliative support. The heterogeneity is affected by multiple factors, such as the patient ‘s functional status, medical accessibility, economic burden, attending physician preferences, etc., which may cause significant interference to OS, thereby weakening OS’ s ability to reflect the direct efficacy of postoperative adjuvant therapy. In contrast, DFS, as an indicator of time from postoperative to first recurrence, is a more sensitive reflection of the actual effectiveness of postoperative adjuvant therapy in delaying tumor recurrence and controlling micrometastases, and is less susceptible to changes in subsequent treatment. Especially in the research environment where there is no unified post-relapse treatment plan, DFS shows higher internal consistency and evaluation value. Therefore, although DFS did not reach statistical significance in some subgroups, its trend improvement still has important clinical guiding significance, which should be paid more attention and interpreted in depth as an important end point index for the evaluation of postoperative adjuvant therapy.

This study also has some limitations. However, several limitations must be acknowledged. Firstly, despite using PSM to minimize confounding bias, the retrospective design cannot exclude the possibility of residual confounding from unmeasured variables. Secondly, there is a certain heterogeneity in adjuvant therapy (TACE times, drug regimen differences, etc.), which may affect the outcome evaluation. Thirdly, the heterogeneity in salvage therapies after recurrence may confound the interpretation of OS, whereas DFS might more directly reflect the effect of the initial adjuvant therapy. Fourthly, our cohort consisted solely of Asian patients, which may limit the generalizability of our findings to other populations due to potential biological and clinical differences. Finally, the inability to separately analyze HAIC due to limited sample size warrants investigation in future studies. In the future, it is necessary to further optimize the individualized adjuvant treatment strategy for high-risk HCC patients through multi-center, prospective randomized controlled studies, combined with molecular typing and immune microenvironment characteristics.

## 5 Conclusion

In summary, postoperative adjuvant therapy can bring significant survival benefits to HCC patients with high-risk recurrence factors. Based on our study, TACE therapy or TKI therapy is recommended for postoperated patients with high AFP levels, multiple tumors, or MVI. For tumors diameter≥5 cm, TACE therapy appears more effective and is therefore preferred. The results of this study provide a strong clinical basis for the development of individualized adjuvant treatment strategies for patients with high-risk recurrent HCC after surgery.

## Data Availability

The original contributions presented in the study are included in the article/supplementary material. Further inquiries can be directed to the corresponding author.
